# The diagnostic accuracy of photopic negative responses evoked by broadband and chromatic stimuli in a clinically heterogeneous population

**DOI:** 10.1007/s10633-023-09956-5

**Published:** 2023-10-27

**Authors:** Shaun M. Leo, Magella M. Neveu, Patrick Yu-Wai-Man, Omar A. Mahroo, Anthony G. Robson

**Affiliations:** 1 Moorfields Eye Hospital, 162 City Road, London, EC1V 2PD UK; 2 Institute of Ophthalmology, University College London, London, UK; 3 Cambridge Centre for Brain Repair and MRC Mitochondrial Biology Unit, Department of Clinical Neurosciences, University of Cambridge, Cambridge, UK; 4 Cambridge Eye Unit, Addenbrooke’s Hospital, Cambridge University Hospital, Cambridge, UK; 5 Section of Ophthalmology, King’s College London, St Thomas’ Hospital Campus, London, UK; 6 Department of Twin Research and Genetic Epidemiology, King’s College London, St Thomas’ Hospital Campus, London, UK; 7 Physiology, Development and Neuroscience, University of Cambridge, Cambridge, UK

**Keywords:** Electroretinography, Optic nerve diseases, Optic neuropathy, Retina, Retinal ganglion cells, Sensitivity and specificity

## Abstract

**Purpose:**

To compare the diagnostic accuracy of the photopic negative response (PhNR) elicited by red-blue (RB) and white-white (WW) stimuli, for detection of retinal ganglion cell (RGC) dysfunction in a heterogeneous clinical cohort.

**Methods:**

Adults referred for electrophysiological investigations were recruited consecutively for this single-centre, prospective, paired diagnostic accuracy study. PhNRs were recorded to red flashes (1.5 cd·s·m^−2^) on a blue background (10 cd·m^−2^) and to white flashes on a white background (the latter being the ISCEV standard LA 3 stimulus). PhNR results were compared with a reference test battery assessing RGC/optic nerve structure and function including optical coherence tomography (OCT) retinal nerve fibre layer thickness and mean RGC volume measurements, fundus photography, pattern electroretinography and visual evoked potentials. Primary outcome measures were differences in sensitivity and specificity of the two PhNR methods.

**Results:**

Two hundred and forty-three participants were initially enrolled, with 200 (median age 54; range 18–95; female 65%) meeting inclusion criteria. Sensitivity was 53% (95% confidence intervals [CI] 39% to 68%) and 62% (95% CI 48% to 76%), for WW and RB PhNRs, respectively. Specificity was 80% (95% CI 74% to 86%) and 78% (95% CI 72% to 85%), respectively. There was a statistically significant difference between sensitivities (*p* = 0.046) but not specificities (*p* = 0.08) of the two methods. Receiver operator characteristic (ROC) area under the curve (AUC) values were 0.73 for WW and 0.74 for RB PhNRs.

**Conclusion:**

PhNRs to red flashes on a blue background may be more sensitive than white-on-white stimuli, but there is no significant difference between specificities. This study highlights the value and potential convenience of using white-on-white stimuli, already used widely for routine ERG assessment.

## Introduction

Routine electrophysiological assessment of optic nerve and macular retinal ganglion cell (RGC) function often involves cortical visual evoked potential (VEP) and pattern electroretinogram (PERG) methods, performed according to well-established standardised protocols [1–3]. There is increasing interest in use of the photopic negative response (PhNR) to assess global [4–7] and focal [8–11] RGC function. The PhNR can be evoked with many different flash strength and wavelength combinations including white flashes on a white background (WW PhNR) and red flashes on blue background (RB PhNR). The International Society for Clinical Electrophysiology of Vision (ISCEV) extended protocol for the PhNR recommends the use of RB stimuli as studies have reported that this yields a larger amplitude response than broadband (WW) stimuli [12–14], with a few exceptions [15–17]. Diagnostic accuracy relates to the ability of a test to discriminate between health and a target condition [18], and previous studies have compared the diagnostic accuracy of different PhNR stimuli using a case–control methodology [14–16, 19–22], with most focussed on the diagnostic and prognostic potential in glaucoma [23–28]. There is a lack of published studies that compare the relative diagnostic accuracy of RB and WW PhNR stimuli without prior knowledge of the diagnosis in a heterogeneous clinical population.

This study investigates the diagnostic accuracy of WW and RB PhNRs, compared with a test battery of clinical tests routinely used in the diagnosis of retinal ganglion cell disease (our target condition). The aim was to test whether WW PhNR stimuli, used routinely to record the ISCEV standard light-adapted full-field (LA 3) ERG [29], are a suitable alternative to RB stimuli for the detection of retinal ganglion cell dysfunction.

## Methods

This was a prospective, paired diagnostic accuracy study conducted at Moorfields Eye Hospital, London, UK. Ethics committee approval was granted by UK National Research Ethics Committee Wales 6 (reference: 20/WA/0300). All eligible adult (18 +) patients attending the Department of Electrophysiology within the recruitment window were identified through the triage of outpatient referrals and invited to participate in the study.

Each participant underwent examinations and electrophysiology according to routine clinical management with an additional PhNR protocol added at the end of electrophysiological testing. Participants were excluded from the study if they met any of the following criteria:

1. Paediatric patients (< 18 years).
2. Declined or were unable to provide consent.
3. Reference tests were unavailable or unrecordable (e.g. undetectable responses due to severe photoreceptor disease).
4. Poor quality test results, e.g. excessive eye movement/blink artefact, muscle tension, mains artefact.

### Procedures

#### Photopic negative responses (index tests)

PhNRs were recorded binocularly using gold foil corneal electrodes with ipsilateral outer canthus reference electrodes, with a ground electrode situated on the forehead. Pupils were pharmacologically dilated using 1% tropicamide (in many cases supplemented with 2.5% phenylephrine hydrochloride). Responses were recorded using an Espion-E3 system (Diagnosys LLC, Lowell, USA). RB PhNR stimuli consisted of red (640 nm) flashes of ≤ 4 ms duration and 1.5 phot cd·s·m^−2^ stimulus strength presented on a blue background (450 nm; 10 phot cd·m^−2^) as specified in the ISCEV extended protocol for the PhNR [12]. The final result for every acquisition was an average of ≥ 15 responses. Amplifier bandwidth was 0.125-300 Hz. Traces exceeding ± 200 μV were automatically rejected as artefactual. WW PhNRs were recorded adhering to the ISCEV ERG standard [29] and consisted of white 3.0 cd·s·m^−2^ flashes on a white 30 cd·m^−2^ background. Amplifier bandwidth was 0.31-500 Hz. All other WW parameters were the same as for the RB PhNR. One eye from each participant was chosen for analysis: either the affected eye in uniocular disease or the left eye when symptoms or pathology were bilateral. The test order was the same for all participants (WW then RB) with approximately the same amount of time between the two tests.

**Fig. 1:**
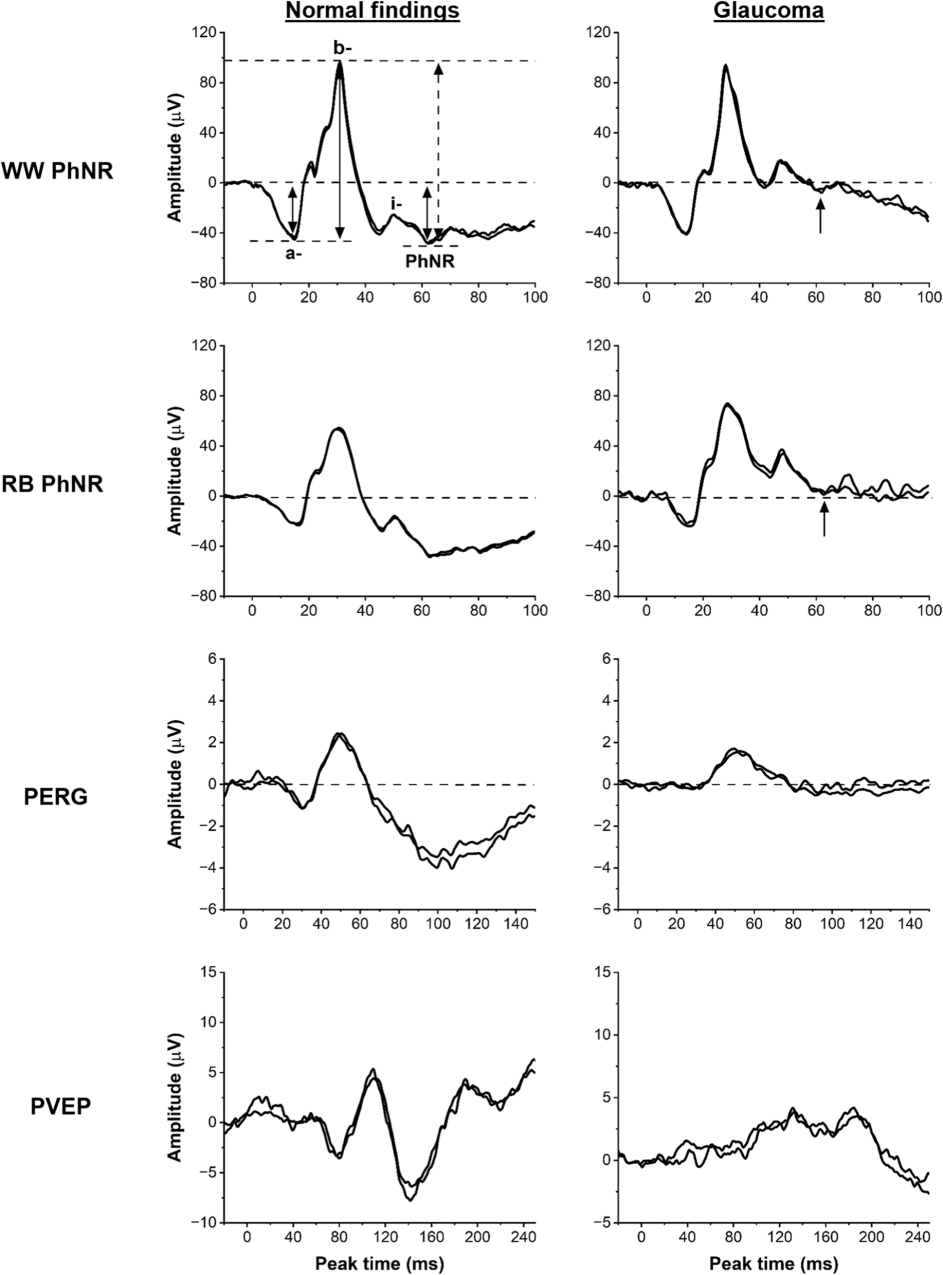
Single eye recordings from two of the study participants. White-white (top row) and red-blue (second row) PhNR recordings and PERGs (third row) and PVEPs (bottom row). Patient 1 (left column), a 68-year-old female, demonstrated no abnormality on any test (normal findings). Patient 2 (right column), a 68-year-old female, was referred with a clinical diagnosis of Glaucoma. Arrows highlight the elevation of the PhNR trough in patient 2, in keeping with generalised retinal ganglion cell dysfunction. The PERG N95:P50 ratio was reduced and the pattern VEP P100 component was delayed and of subnormal amplitude. The main PhNR waveform components are highlighted in the top left panel. Vertical black lines show a- and b-wave amplitude measurements, and PhNR amplitude measurements from baseline. The dashed black line represents the PhNR measured from the peak of the b-wave as used to calculate the PhNR-to-b-wave ratio

The top right panel in Fig. 1 highlights the main waveform components of the photopic negative response. The PhNR was measured from the baseline to the deepest trough of the negativity that followed the b-wave, either before or after the i-wave. Additionally, the PhNR-to-b-wave ratio (PhNR:b) was calculated after measuring the PhNR from the peak of the b-wave to the deepest trough that followed.

#### Reference tests

As no single gold standard test of RGC function exists to directly compare against the PhNRs, a battery of reference tests was used. This consisted of the pattern electroretinogram (PERG) [1] and pattern visual evoked potentials (PVEP) [2], often performed together as part of routine test protocols in the electrophysiology clinic. Fundus photography (Optos plc, Dunfermline, UK) and optical coherence tomography (OCT) (Spectralis Heidelberg Engineering Ltd, Heidelberg, Germany) measures of retinal nerve fibre layer thickness (RNFL) and mean ganglion cell layer volume were assessed as part of routine clinical assessment. Additionally, relevant clinical and family history was recorded from all participants during their visit, as part of routine clinical care. All reference tests were performed according to current clinical standards.

Due to the nature of the study population, clinical judgement was required to assign the participants into groups according to evidence of RGC pathology. All participants with a reduced PERG N95:P50 ratio were included in the ‘evidence of RGC pathology’ group, as were those with OCT evidence of RNFL thinning. In all other cases, at least two abnormal reference tests were required. In cases where only one of the reference tests were abnormal, the clinical notes were reviewed and those with an established diagnosis of optic neuropathy were included, e.g. glaucoma and abnormal VEP.

The investigator interpreting the index tests (SL) was masked to the result of the reference tests. Conversely, investigators (AR and MN) interpreting the reference tests were masked to the results of the index tests.

### Definition of clinically significant retinal ganglion cell dysfunction

Significant RGC dysfunction was defined using local reference ranges from the control PhNR dataset. The lower limit (5th centile) of the reference ranges for amplitudes of the RB and WW PhNRs was 18.4 µV and 12.8 µV, respectively. Reference test results (including PERG and VEP) were compared with local reference ranges and were analysed by experienced electrophysiologists (MN; AGR). Participants were then categorised into either the ‘no evidence of RGC pathology’ group or the ‘evidence of RGC pathology’ group.

The primary outcome was the difference between the sensitivities and specificities of WW and RB PhNRs, derived using paired contingency tables. Secondary outcomes were the difference between the group amplitudes of the two PhNR types, PhNR positive and negative predictive values and area under the receiver operating characteristic (ROC) curves.

### Statistical methods

Descriptive statistics were performed on PhNR amplitudes. The distribution of the data was evaluated using the Shapiro–Wilk test, and the RB and WW groups were planned to be compared using either a two-tailed t test or the nonparametric Mann–Whitney U test if the distribution of the data was not Gaussian. Results were considered statistically significant if *p* < 0.05. Sensitivities and specificities were calculated using paired contingency tables [30]. McNemar tests were used to compare the estimated sensitivities, specificities, positive predictive values (PPVs) and negative predictive values (NPVs) of the WW and RB PhNRs [31, 32]. The relationship between true (sensitivity) and false (1-sensitivity) positive rates across a range of cut-off points was investigated using ROC curves, and test performance was measured using the area under the ROC curve (AUC) [33]. Where applicable, 95% confidence intervals (CI) were applied to the above results. Analyses were conducted using R version 3.6.3 (R Foundation for Statistical Computing, Vienna, Austria) and OriginPro 2019 (OriginLab, Northampton, USA).

### Sample size calculation

Sample size was calculated following the methods of McCray et al. [34]. Prevalence of RGC dysfunction within the study population was estimated to be 50%. A power of 80% at an alpha level of 0.05 was used for the calculation, giving an estimated minimum sample size of 152 participants.

## Results

### Baseline demographics

**Fig. 2:**
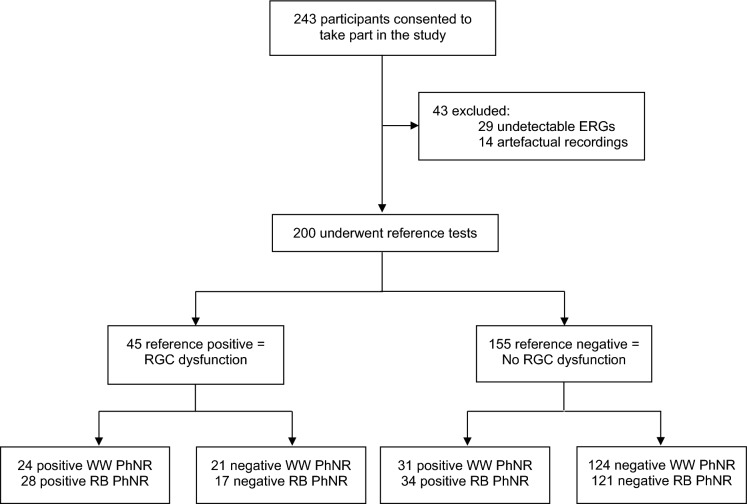
Flow of all participants through the study categorised with the baseline-to-trough PhNR

Recruitment was between March 2021 and February 2022 and included 243 consecutive patients who provided consent to take part in the study. The flow of participants through the study is outlined in Fig. 2. Twenty-nine participants had undetectable or residual full-field ERGs due to severe generalised retinal dysfunction and were excluded from the analysis. RB PhNR recordings from 14 participants had excessive levels of blink/eye movement artefact precluding reliable quantification and were excluded from the analysis. Results from the remaining 200 participants were analysed (completion rate 82%). The median age of participants was 54 years (range 18–95), and 129 participants (65%) were female. Table 1 summarises the characteristics of the participants who were categorised as either having evidence of RGC dysfunction or no evidence of RGC dysfunction. Clinical findings and history, VEP and PERG results were available for all participants. OCT RNFL was performed on 59% of participants, and mGCL volume results were available for 90%. Table 2 summarises the results of the reference tests in those in the ‘evidence of RGC pathology’ group. There were no significant time delays in conducting any of the investigations, and no adverse events occurred as a result of any of the tests.

**Table 1:** Baseline demographics and characteristics of all participants recruited to the study

Patient characteristics	Total no. (%)
*RGC dysfunction (reference tests positive)*	
Median age, (range)	54 (18–77)
Women	23 (51.1)
*Diagnosis*	
Hereditary optic neuropathy	7 (15.6)
Glaucoma	7 (15.6)
Disc drusen	2 (4.4)
Multiple sclerosis	3 (6.7)
Meningioma	2 (4.4)
Optic atrophy (cause unknown)	7 (15.6)
Unconfirmed	17 (37.7)
*No RGC dysfunction (reference tests negative)*	
Median age, (range)	54 (18–95)
Women	91 (58.7)
*Diagnosis*	
Birdshot chorioretinopathy	22 (14.2)
Uveitis	8 (5.2)
Autoimmune retinopathy	12 (7.7)
Acute zonal occult outer retinopathy	7 (4.5)
Normal findings/functional visual loss	24 (15.5)
Macular dysfunction	27 (17.4)
ABCA4 retinopathy	4 (2.6)
Hydroxychloroquine toxicity	4 (2.6)
Vitamin A deficiency	1 (0.6)
Hereditary retinal dystrophy	13 (8.4)
Visual snow/migraines	3 (1.9)
White dot syndrome	2 (1.3)
Unconfirmed	28 (18.1)

Unconfirmed refers to participants without an established clinical diagnosis at the time of publication

**Table 2:** Abnormal reference test results in those in the ‘reference positive’ group

Diagnosis (No. of patients)	No. with abnormal findings			
	PERG N95:P50	PVEP	RNFL	mGCL volume
Hereditary optic neuropathy (7)	5/7	7/7	7/7	7/7
Glaucoma (7)	3/7	6/7	**5/6**	5/7
Disc drusen (2)	2/2	2/2	**0**	**1/1**
Multiple sclerosis (3)	2/3	3/3	3/3	3/3
Meningioma (2)	1/2	2/2	**1/1**	2/2
Optic atrophy (cause unknown) (7)	7/7	7/7	7/7	7/7
Unconfirmed (17)	14/17	10/17	**4/11**	**9/14**

Bold text highlights when reference test results were unavailable for some participants

### Photopic negative response amplitudes

**Fig. 3:**
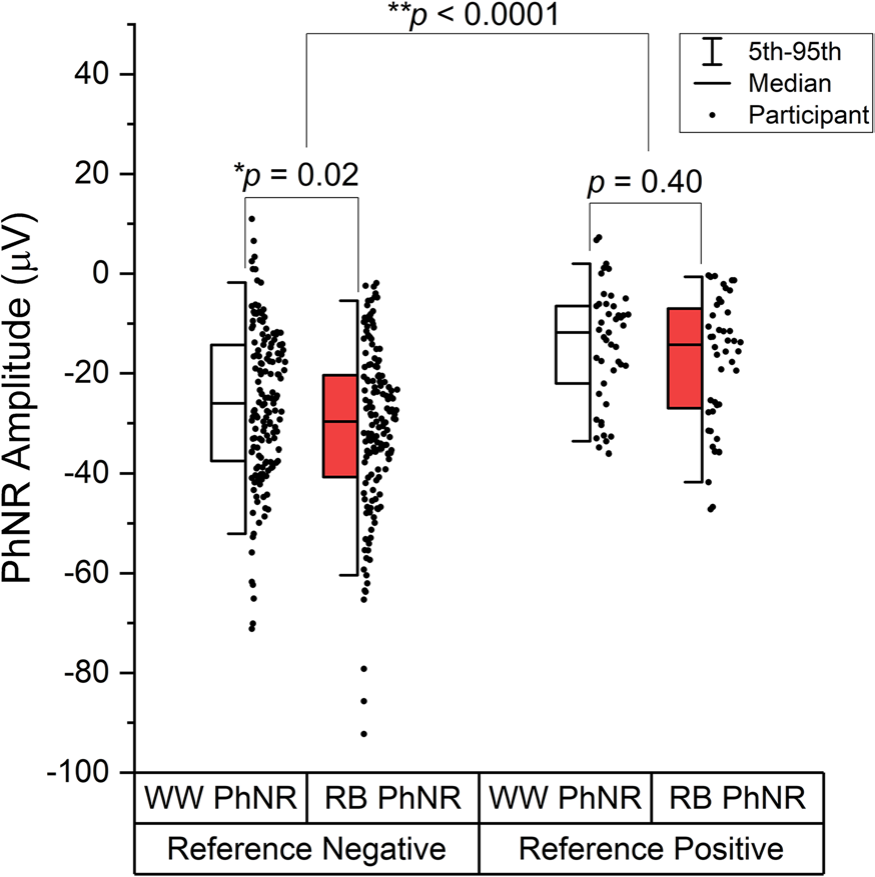
Half box plots showing the distribution of PhNR amplitudes for all patients grouped according to the reference test result. Individual data points from each participant are shown to the right of each box. Whiskers show 5th and 95th centiles. Boxes show the 25th centile, median, and 75th centile

Shapiro–Wilk tests determined that PhNR amplitudes were not drawn from a normally distributed population, and therefore the nonparametric Mann–Whitney U test was used. Amplitude findings are presented in Fig. 3. The median baseline-to-trough amplitudes of RB and WW PhNRs were 27.3 µV and 22.6 µV, respectively. The mean baseline-to-trough amplitudes of RB and WW PhNRs were 28.2 µV and 23.7 µV, respectively. The minimum and maximum, median and 5th and 95th centiles of RB PhNR amplitudes were larger than those of the WW PhNR, and there was a statistically significant difference between the amplitudes of RB and WW PhNRs in the participants without any RGC pathology (*p* = 0.02). There was no significant difference between the amplitudes of RGC pathology positive RB and RGC pathology positive WW PhNRs (*p* = 0.40). There was a highly significant difference between the amplitudes of all participants with RGC pathology grouped together versus the participants with no evidence of RGC pathology (*p* < 0.0001).

### Estimates of diagnostic accuracy

The diagnostic performance of WW PhNRs and RB PhNRs were compared using contingency tables**.** A summary of the results is displayed in Table 3. Forty-five patients had evidence of RGC dysfunction giving an overall prevalence of 23% in the study cohort. An example of the electrophysiological findings from two participants is shown in Fig. 1.

**Table 3:** Diagnostic accuracy measures for all participants (N = 200)

	RB PhNR (95% CI)	WW PhNR (95% CI)	Difference/Test ratio (95% CI)	*p* value
*From baseline*				
Sensitivity	0.62 (0.48–0.76)	0.53 (0.39–0.68)	− 0.09 (− 0.17–(− 0.01))	0.046*
Specificity	0.78 (0.72–0.85)	0.80 (0.74–0.86)	0.02 (0.00–0.04)	0.08
PPV	0.45 (0.33–0.58)	0.44 (0.31–0.57)	1.04 (0.93–1.15)	0.52
NPV	0.88 (0.82–0.93)	0.86 (0.80–0.91)	1.03 (1.00–1.05)	0.08
*PhNR:b-wave ratio*				
Sensitivity	0.49 (0.34–0.64)	0.40 (0.26–0.54)	− 0.09 (− 0.17–(− 0.01))	0.046*
Specificity	0.88 (0.83–0.93)	0.89 (0.84–0.94)	0.01 (-0.01–0.03)	0.16
PPV	0.54 (0.38–0.69)	0.51 (0.35–0.68)	1.04 (0.92–1.18)	0.50
NPV	0.86 (0.80–0.91)	0.84 (0.78–0.89)	1.02 (1.00–1.05)	0.07

^*^p < 0.05 considered statistically significant

#### Baseline-to-trough

The sensitives of WW and RB PhNRs were 53% (95% CI 39% to 68%) and 62% (95% CI 48% to 76%), respectively. The difference between the sensitivities was -9% (95% CI − 17% to − 1%). Specificities were 80% (95% CI 74% to 86%) and 78% (95% CI 72% to 85%), for WW and RB PhNRs, respectively. The difference between the specificities was 2% (95% CI 0% to 4%). Positive predictive values of WW and RB PhNRs were 44% (95% CI 31% to 57%) and 45% (95% CI 33% to 58%), respectively. Negative predictive values were 86% (95% CI 80% to 91%) and 88% (95% CI 82% to 93%), for WW and RB PhNRs, respectively. McNemar’s test found a statistically significant difference between the sensitivities of WW and RB PhNRs (*p* = 0.046). There was no statistically significant difference between the specificities of WW and RB PhNRs (*p* = 0.08). There were no statistically significant differences between the WW and RB positive predictive values (*p* = 0.52) or the WW and RB negative predictive values (*p* = 0.08).

#### PhNR:b-wave ratio

PhNR:b-wave ratios were also analysed, and detailed results are given in Table 3. Compared with PhNR amplitudes, ratio values reduced the sensitivity to RGC dysfunction from 53 to 40% and from 62 to 49%, for the WW and RB PhNR, respectively. Use of the PhNR:b-wave ratio increased specificity from 80 to 89% and from 78 to 88% for the WW and RB responses, respectively.

The WW PhNR amplitudes were abnormal, and the PhNR:b-wave ratio was normal in nine of 45 cases and the ratio solely abnormal in three cases. For RB, PhNR amplitudes were abnormal, and the PhNR:b-wave ratio was normal in seven of 45 cases and the ratio solely abnormal in one case.

#### ‘Normal’ LA 3 a- and b-waves

In this sub-analysis, participants with an LA 3 a- or b-wave outside of the control reference range (amplitude and peak time) were excluded. This was to examine whether the diagnostic accuracies (outlined above) were influenced by other, non-RGC pathologies. Fifty-one participants met these criteria and were excluded leaving a total of 149 (38 with RGC pathology). The results are summarised in Table 4. The sensitivities of WW and RB PhNRs were 50% and 61%, respectively. The difference between the sensitivities was − 11%. Specificities were 94% and 90%. The difference between the specificities was 4%. There was a statistically significant difference between the sensitivities and the specificities of WW and RB PhNRs (both *p* = 0.046).

**Table 4:** Diagnostic accuracy measures for patients with normal LA 3 a- and b-waves (N = 149)

	RB PhNR (95% CI)	WW PhNR (95% CI)	Difference/Test ratio (95% CI)	*p* value
*From baseline:*				
Sensitivity	0.61 (0.45–0.76)	0.50 (0.34–0.66)	− 0.11 (− 0.20–(− 0.01))	0.046*
Specificity	0.90 (0.85–0.96)	0.94 (0.89–0.98)	0.04 (0.00–0.07)	0.046*
PPV	0.68 (0.52–0.83)	0.73 (0.56–0.90)	0.93 (0.81–1.06)	0.27
NPV	0.87 (0.81–0.93)	0.85 (0.78–0.91)	1.03 (1.00–1.06)	0.1
*PhNR:b-wave ratio:*				
Sensitivity	0.47 (0.32–0.63)	0.32 (0.17–0.46)	-0.16 (-0.27-(-0.04)	0.01*
Specificity	0.93 (0.88–0.98)	0.97 (0.94–1.00)	0.05 (0.01–0.08)	0.03*
PPV	0.69 (0.52–0.87)	0.80 (0.60–1.00)	0.87 (0.70–1.10)	0.18
NPV	0.84 (0.77–0.90)	0.81 (0.74–0.87)	1.04 (1.00–1.08)	0.045*

^*^p < 0.05 considered statistically significant

#### ROC curves

**Fig. 4:**
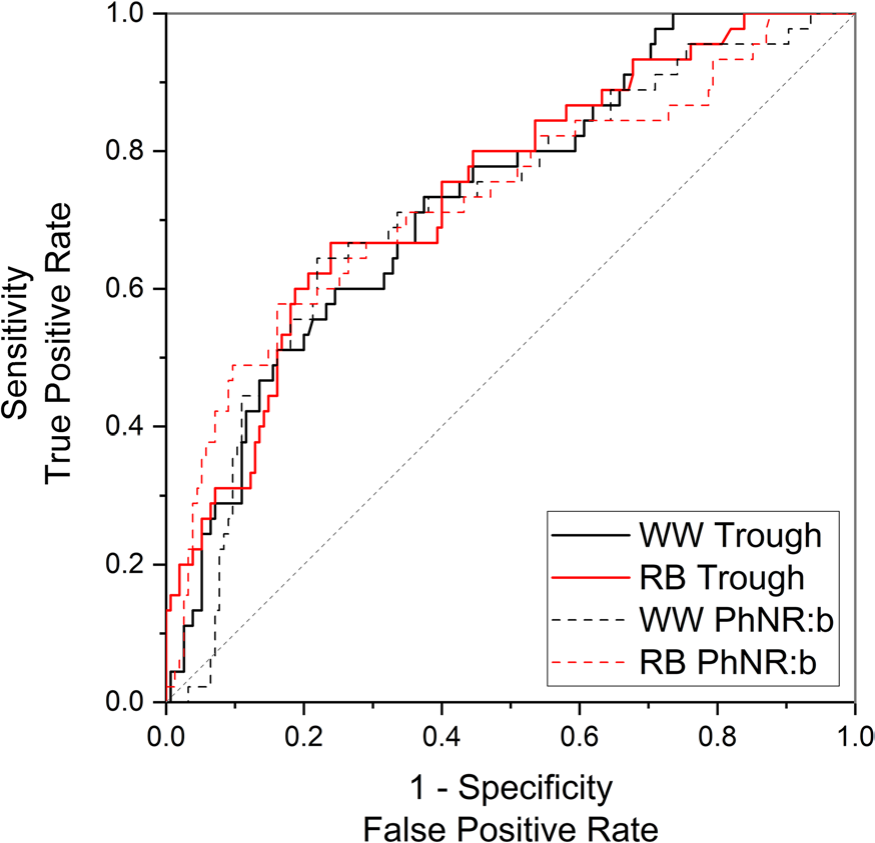
Receiver operator characteristic curves for WW and RB PhNRs from all patients (N = 200)

**Fig. 5:**
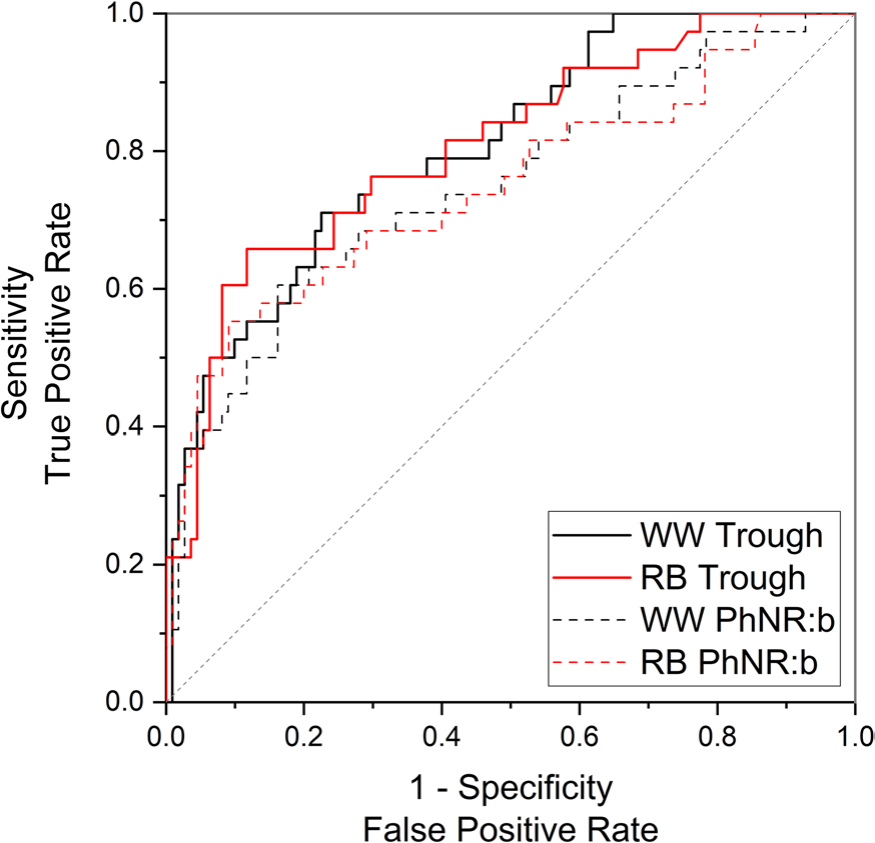
Receiver operator characteristic curves for WW and RB PhNRs from patients with normal a- and b-waves (N = 149)

Figures 4 and 5 display ROC curves for the PhNRs with sensitivity plotted against 1-specificity. The output values from the ROC curves are summarised in Table 5. The AUC value for the WW PhNR was 0.73 (95% CI 0.65 to 0.82; *p* < 0.001). The RB PhNR AUC was 0.74 (95% CI 0.66 to 0.82; *p* < 0.001). The criterion values (optimal cut-offs where sensitivity and specificity values are closest to the AUC value and have a minimal difference between them [35]) for the WW and RB PhNR amplitudes were 17.7 µV and 23.5 µV, respectively. In participants with normal LA 3 a- and b-waves, ROC AUC values increased to 0.81 for both WW and RB PhNRs.

**Table 5:** Receiver operator characteristic curve summary for all participants

	RB Trough (95% CI)	WW Trough (95% CI)	RB PhNR:b (95% CI)	WW PhNR:b (95% CI)
*All participants:*				
AUC	0.74 (0.66–0.82)	0.73 (0.65–0.82)	0.73 (0.65–0.81)	0.72 (0.63–0.81)
*p* value	7 × 10^–7^*	2 × 10^–6^*	2 × 10^–6^*	8 × 10^–6^*
Criterion value	23.5 µV	17.7 µV	0.93	0.87
Sensitivity	0.67	0.67	0.67	0.67
Specificity	0.67	0.67	0.67	0.66
*Abnormal a- and b-waves excluded:*				
AUC	0.81 (0.73–0.88)	0.81 (0.73–0.89)	0.75 (0.67–0.83)	0.75 (0.67–0.83)
*p* value	2 × 10^–8^*	2 × 10^–8^*	5 × 10^–6^*	5 × 10^–6^*
Criterion value	27.5 µV	21.5 µV	0.94	0.88
Sensitivity	0.71	0.71	0.68	0.68
Specificity	0.71	0.72	0.68	0.69

^*^p < 0.05 considered statistically significant

## Discussion

This study examines the diagnostic accuracy of PhNRs in the largest patient cohort to date, by comparison with multi-modal assessments of optic nerve structure and standardised electrophysiological tests of function. Uniquely, the diagnostic accuracy of PhNRs evoked by chromatic and broadband stimuli is compared in a heterogeneous rather than a case-controlled clinical population, providing a robust estimation of sensitivity and specificity more applicable to the general patient population. The potential of using a widely available ISCEV standard full-field ERG protocol to assess retinal ganglion cell function is examined.

This study showed that PhNR amplitudes are larger when elicited by the ISCEV-recommended RB [12] rather than ISCEV standard (LA 3 ERG) WW [29] stimuli, consistent with several previous comparisons of chromatic and broadband stimuli [14, 19]. It has been suggested that a chromatic stimulus may preferentially stimulate a single subtype of cone, reducing the amount of spectral antagonism in the receptive fields of the RGCs [18]. It is noted, however, that PhNR amplitudes are only minimally influenced by the chromaticities of the flash and background when stimuli are expressed in photopic photometric terms [15, 36] and photopically matched, with one report that RB are larger than WW PhNRs only at higher flash strengths (3 phot cd·s·m^−2^). In the present study, the statistically significant amplitude difference between PhNRs seen in those without RGC pathology was not apparent in patients with RGC dysfunction. The possibility that dysfunction disproportionately attenuates chromatic-evoked responses and that this may be related to disease type and severity warrants further investigation.

The study shows that the RB PhNR has a higher sensitivity than the WW PhNR for the detection of RGC dysfunction (62% vs 53%) and that this difference is statistically significant. The confidence intervals of the difference between the sensitivities (95% CI − 17% to − 1%) further support this finding as the range does not encompass zero [37]. These findings suggest that the RB PhNR stimulus is better able to detect RGC pathology than the WW stimulus in a heterogeneous clinical population. Estimations of specificity did not significantly differ for the two stimuli (78% and 80% for RB and WW), suggesting that both methods can identify unaffected individuals to a similar degree. The findings suggest that the RB PhNR is more likely to detect RGC disease in affected individuals, but as the specificities of the PhNR stimuli are equivalent, a positive finding in the WW PhNR is just as likely to be a true positive as a positive result from the RB PhNR. This is an important finding in the context of clinical practice, as RB PhNR protocols are not fully standardised and are less widely available than WW PhNRs, as the latter form part of the ISCEV standard full-field LA 3 ERG, used routinely to assess retinal function.

In our cohort, the PhNR:b-wave ratio lowered the sensitivity of the test when compared with the PhNR amplitude measure. Perhaps not surprisingly, specificity increased as the ratio takes into account ERG amplitude variability and the possibility of ERG b-wave attenuation, e.g. due to retinal (non-RGC) pathology. In support of this, the specificity of both measurements was equivalent when cases with an abnormal LA 3 ERG a- and b-wave were excluded from the comparison, highlighting the importance of considering retinal function and light-adapted ERG a- and b-waves when interpreting the PhNR clinically.

There were no significant differences in the ROC AUCs of WW and RB PhNRs measured with the amplitude or ratio methods, consistent with some previous studies on patients with glaucoma [10, 38]. However, differences have also been reported; Cvenkel et al., [23] found that the PhNR amplitude provided significantly larger AUCs than the ratio for both suspect and early glaucoma, and Preiser et al. [39] found that the PhNR:b-wave ratio yielded higher AUC values than amplitude measures in pre-perimetric but not manifest glaucoma. These conflicting reports may relate to different methods and individual differences, and it may be prudent to consider both measurements (of the same waveform) for diagnostic or monitoring purposes.

In this study, the estimated area under the ROC curves for RB and WW PhNRs suggests only a modest level of diagnostic accuracy, as defined by ROC reporting guidelines [33]. A contributory factor may be the heterogeneity and diversity of the clinical patients examined, with different disorders and at different stages of disease severity. The overall diagnostic accuracies of RB and WW PhNRs estimated by AUCs were equivalent (0.74 vs 0.73 for RB and WW), a finding in contrast with some prior reports. Sustar et al. [14] reported AUC values of 0.97 and 0.74 for RB and WW stimuli, respectively, and Banerjee et al. [21] also reported higher RB AUC values of 0.90 compared with 0.76 for WW stimuli. Hara et al. [16] calculated AUCs for a range of photopically matched RB and WW stimuli and found that the 3.0 cd·s·m^−2^ RB stimulus provided the best diagnostic accuracy overall (AUC = 0.94); the best WW PhNR stimulus was obtained with a 2.0 cd·s·m^−2^ flash (AUC = 0.88). In these studies, examinations of pathology were restricted to patients with glaucoma, which may account for some of the divergence with our findings; nonetheless, the AUC evidence suggests that RB stimuli may have better overall diagnostic accuracy than WW stimuli for the detection of glaucoma.

Our heterogeneous group of patients with RGC dysfunction included seven with mitochondrial optic neuropathies (Leber hereditary optic neuropathy (LHON) and autosomal dominant optic atrophy (DOA)). These disorders primarily affect the papillomacular bundle, with relative sparing of peripheral RGC axons [40], particularly in the early stages of the disease process. As the full-field PhNR is a global measure of RGC function it is likely to be less sensitive to focal/central RGC dysfunction than the PERG or the focal PhNR. Majander et al. [41] reported that the majority of full-field PhNR responses from a cohort of patients with LHON were normal or near the lower limit of normal. Likewise, Miyata et al. [42] found only mildly abnormal/borderline reductions in the full-field PhNR in patients with DOA. Tamada et al. [43] investigated the ability of focal and full-field PhNRs to detect optic nerve atrophy and found that focal PhNRs were more sensitive to damage in eyes with a central visual field defect. The inclusion of patients with both central and diffuse RGC damage in the present study is likely another reason that the estimations of sensitivity and specificity are lower than previous reports.

There are some limitations to this study. In our preliminary experiments, a stimulus–response series was used to determine the optimal RB PhNR stimuli to compare against the broadband PhNR. An aim was to assess the diagnostic accuracy of the ISCEV LA 3.0 ERG PhNR component, which may be of lower diagnostic accuracy compared with optimised WW stimuli. Our aim to compare the LA 3 WW PhNR against the RB PhNR from the ISCEV extended protocol also meant that there was a slight difference between the amplifier bandwidths of the two protocols. The high-pass filter of the WW PhNR was 0.31 Hz, while the high pass of the RB PhNR was 0.13 Hz. As the PhNR is a relatively slow frequency response, this difference may have contributed to the smaller PhNR amplitude that we reported for the WW PhNR. Another limitation is that there is no single gold standard test of retinal ganglion cell dysfunction and therefore multiple tests combined into a test battery were needed to establish the diagnostic status of the participants. Not all participants performed all of the reference tests; this means that not all participants were compared to an identical reference standard, raising the possibility of some verification bias [44]. The group comparison showed that sensitivity to RGC dysfunction was higher for PhNR amplitude than for the PhNR:b-wave ratio, but it is acknowledged that the ratio is likely to be informative in cases of dual pathology, e.g. to judge the severity of optic nerve/RGC dysfunction in the presence of retinopathy (manifest as ERG b-wave and ‘downstream’ PhNR reduction). Strengths of the study include that it was appropriately powered and based on an ethnically diverse and relatively large number of patients, from a consecutive sample referred to an electrophysiology department rather than a case–control population. This enabled a robust estimation of sensitivity and specificity generalisable to the general population.

The PhNR yields moderate levels of diagnostic accuracy for the detection of retinal ganglion cell dysfunction in a heterogeneous clinical cohort. PhNRs evoked by red flashes on a blue background are more sensitive to dysfunction than white-on-white stimuli, but there is no significant difference between the relative specificities of the two PhNR methods. The study highlights the value and potential convenience of using the WW stimulus, already used widely for routine ERG assessment of retinal function.

## References

[1] Bach M, Brigell MG, Hawlina M, Holder GE, Johnson MA, McCulloch DL (2013). ISCEV standard for clinical pattern electroretinography (PERG): 2012 update. Doc Ophthalmol.

[2] Odom JV, Bach M, Brigell M, Holder GE, McCulloch DL, Mizota A (2016). ISCEV standard for clinical visual evoked potentials: (2016 update). Doc Ophthalmol.

[3] Jurkute N, Robson AG (2021). Electrophysiology in neuro-ophthalmology. Handb Clin Neurol.

[4] Robson AG, Nilsson J, Li S, Jalali S, Fulton AB, Tormene AP (2018). ISCEV guide to visual electrodiagnostic procedures. Doc Ophthalmol.

[5] Marmoy OR, Viswanathan S (2021). Clinical electrophysiology of the optic nerve and retinal ganglion cells. Eye.

[6] Constable PA, Lee IO, Marmolejo-Ramos F, Skuse DH, Thompson DA (2021). The photopic negative response in autism spectrum disorder. Clin Exp Optom.

[7] Moghimi P, Jimenez NT, McLoon LK, Netoff TI, Lee MS, MacDonald A (2020). Electoretinographic evidence of retinal ganglion cell -dependent function in schizophrenia. Schizophr Res.

[8] Al-Nosairy KO, Prabhakaran GT, Pappelis K, Thieme H, Hoffmann MB (2020). Combined multi-modal assessment of glaucomatous damage with electroretinography and optical coherence tomography/angiography. Transl Vis Sci Technol.

[9] Kondo M, Kurimoto Y, Sakai T, Koyasu T, Miyata K, Ueno S (2008). Recording focal macular photopic negative response (PhNR) from monkeys. Invest Ophthalmol Vis Sci.

[10] Machida S, Toba Y, Ohtaki A, Gotoh Y, Kaneko M, Kurosaka D (2008). Photopic negative response of focal electoretinograms in glaucomatous eyes. Invest Ophthalmol Vis Sci.

[11] Morny EKA, Margrain TH, Binns AM, Votruba M (2015). Electrophysiological ON and OFF responses in autosomal dominant optic atrophy. Invest Ophthalmol Vis Sci.

[12] Frishman L, Sustar M, Kremers J, McAnany JJ, Sarossy M, Tzekov R (2018). ISCEV extended protocol for the photopic negative response (PhNR) of the full-field electroretinogram. Doc Ophthalmol.

[13] Rangaswamy NV, Digby B, Harwerth RS, Frishman LJ (2005). Optimizing the spectral characteristics of a ganzfeld stimulus used for eliciting the photopic negative response (PhNR). Investig Ophthalmol Vis Sci.

[14] Sustar M, Cvenkel B, Brecelj J (2009). The effect of broadband and monochromatic stimuli on the photopic negative response of the electroretinogram in normal subjects and in open-angle glaucoma patients. Doc Ophthalmol.

[15] Kremers J, Jertila M, Link B, Pangeni G, Horn FK (2012). Spectral characteristics of the PhNR in the full-field flash electroretinogram of normals and glaucoma patients. Doc Ophthalmol.

[16] Hara Y, Machida S, Ebihara S, Ishizuka M, Tada A, Nishimura T (2020). Comparisons of photopic negative responses elicited by different conditions from glaucomatous eyes. Jpn J Ophthalmol.

[17] Wakili N, Horn FK, Jünemann AG, Nguyen NX, Mardin CY, Korth M (2008). The photopic negative response of the blue-on-yellow flash-electroretinogram in glaucomas and normal subjects. Doc Ophthalmol.

[18] Šimundić A-M (2009). Measures of diagnostic accuracy: basic definitions. EJIFCC.

[19] Rangaswamy NV, Shirato S, Kaneko M, Digby BI, Robson JG, Frishman LJ (2007). Effects of spectral characteristics of ganzfeld stimuli on the photopic negative response (PhNR) of the ERG. Invest Ophthalmol Vis Sci.

[20] Shen X, Huang L, Fan N, He J (2013). Relationship among photopic negative response, retinal nerve fiber layer thickness, and visual field between normal and POAG Eyes. ISRN Ophthalmol.

[21] Banerjee A, Khurana M, Sachidanandam R, Sen P (2019). Comparison between broadband and monochromatic photopic negative response in full-field electroretinogram in controls and subjects with primary open-angle glaucoma. Doc Ophthalmol.

[22] Wilsey L, Gowrisankaran S, Cull G, Hardin C, Burgoyne CF, Fortune B (2017). Comparing three different modes of electroretinography in experimental glaucoma: diagnostic performance and correlation to structure. Doc Ophthalmol.

[23] Cvenkel B, Sustar M, Perovsek D (2017). Ganglion cell loss in early glaucoma, as assessed by photopic negative response, pattern electroretinogram, and spectral-domain optical coherence tomography. Doc Ophthalmol.

[24] Kita Y, Hollo G, Saito T, Momota Y, Kita R, Tsunoda K (2020). RETeval portable electroretinogram parameters in different severity stages of glaucoma. J Glaucoma.

[25] Huang L, Shen X, Fan N, He J (2012). Clinical application of photopic negative response of the flash electroretinogram in primary open-angle Glaucoma. Eye Sci.

[26] Machida S, Tamada K, Oikawa T, Gotoh Y, Nishimura T, Kaneko M (2011). Comparison of photopic negative response of full-field and focal electroretinograms in detecting glaucomatous eyes. J Ophthalmol.

[27] Machida S, Gotoh Y, Toba Y, Ohtaki A, Kaneko M, Kurosaka D (2008). Correlation between photopic negative response and retinal nerve fiber layer thickness and optic disc topography in glaucomatous eyes. Invest Ophthalmol Vis Sci.

[28] Machida S, Tamada K, Oikawa T, Yokoyama D, Kaneko M, Kurosaka D (2010). Sensitivity and specificity of photopic negative response of focal electoretinogram to detect glaucomatous eyes. Br J Ophthalmol.

[29] Robson AG, Frishman LJ, Grigg J, Hamilton R, Jeffrey BG, Kondo M (2022). ISCEV Standard for full-field clinical electroretinography (2022 update). Doc Ophthalmol.

[30] Hayen A, Macaskill P, Irwig L, Bossuyt P (2010). Appropriate statistical methods are required to assess diagnostic tests for replacement, add-on, and triage. J Clin Epidemiol.

[31] McNemar Q (1947). Note on the sampling error of the difference between correlated proportions or percentages. Psychometrika.

[32] Moskowitz CS, Pepe MS (2006). Comparing the predictive values of diagnostic tests: sample size and analysis for paired study designs. Clin Trials.

[33] Florkowski CM (2008). Sensitivity, specificity, receiver-operating characteristic (ROC) curves and likelihood ratios: communicating the performance of diagnostic tests. Clin Biochem Rev.

[34] McCray GPJ, Titman AC, Ghaneh P, Lancaster GA (2017). Sample size re-estimation in paired comparative diagnostic accuracy studies with a binary response. BMC Med Res Methodol.

[35] Unal I (2017). Defining an optimal cut-point value in ROC analysis: an alternative approach. Comput Math Methods Med.

[36] Miyata K, Ueno S, Kondo M, Koyasu T, Terasaki H (2008). Comparison of photopic negative responses elicited by red and white xenon flashes in monkeys. Jpn J Ophthalmol.

[37] Sim J, Reid N (1999). Statistical inference by confidence intervals: issues of interpretation and utilization. Phys Ther.

[38] Kirkiewicz M, Lubinski W, Penkala K (2016). Photopic negative response of full-field electroretinography in patients with different stages of glaucomatous optic neuropathy. Doc Ophthalmol.

[39] Preiser D, Lagreze WA, Bach M, Poloschek CM (2013). Photopic negative response versus pattern electroretinogram in early glaucoma. Invest Ophthalmol Vis Sci.

[40] Yu-Wai-Man P, Griffiths PG, Hudson G, Chinnery PF (2009). Inherited mitochondrial optic neuropathies. J Med Genet.

[41] Majander A, Robson AG, Joao C, Holder GE, Chinnery PF, Moore AT (2017). The pattern of retinal ganglion cell dysfunction in Leber hereditary optic neuropathy. Mitochondrion.

[42] Miyata K, Nakamura M, Kondo M, Lin J, Ueno S, Miyake Y (2007). Reduction of oscillatory potentials and photopic negative response in patients with autosomal dominant optic atrophy with OPA1 mutations. Invest Ophthalmol Vis Sci.

[43] Tamada K, Machida S, Yokoyama D, Kurosaka D (2009). Photopic negative response of full-field and focal macular electroretinograms in patients with optic nerve atrophy. Jpn J Ophthalmol.

[44] Whiting P, Rutjes AW, Reitsma JB, Glas AS, Bossuyt PM, Kleijnen J (2004). Sources of variation and bias in studies of diagnostic accuracy: a systematic review. Ann Intern Med.

